# Clinical Evaluation of Response to Octreotide and Chemotherapy in High-Grade Malignant Neuroendocrine Tumors and Promising In Vitro Preclinical Results with Pasireotide

**DOI:** 10.3390/medicina60071039

**Published:** 2024-06-25

**Authors:** Kevin Doello, Maria Angeles Chico, Francisco Quiñonero, Raúl Ortiz, Jose Prados, Cristina Mesas, Consolación Melguizo

**Affiliations:** 1 Medical Oncology Service, Virgen de las Nieves Hospital, 18014 Granada, Spain; kevindoello@gmail.com; 2 Instituto de Investigación Biosanitaria de Granada (ibs.GRANADA), 18014 Granada, Spain; mchico@ugr.es (M.A.C.); roquesa@ugr.es (R.O.); 3 Institute of Biopathology and Regenerative Medicine (IBIMER), Biomedical Research Center (CIBM), 18100 Granada, Spain; fjquinonero@ugr.es (F.Q.); melguizo@ugr.es (C.M.); 4 Department of Anatomy and Embryology, University of Granada, 18071 Granada, Spain

**Keywords:** carcinoma, pasireotide, somatostatin receptors, neuroendocrine tumors

## Abstract

*Background and Objectives*: High-grade malignant neuroendocrine tumors (G3 NETs) and neuroendocrine carcinomas (NECs) are characterized by rapid proliferation, high metastatic capacity, and strong expression of somatostatin receptors (SSTRs). We aimed to analyze the presence of SSTRs in NET G3 and NEC, and to correlate their expression with the use of octreotide and pasireotide. *Materials and Methods*: For this purpose, we first performed a retrospective study of G3 NET and NEC patients, which included the determination of SSTR expression and response to octreotide treatment. Second, we selected the H69 small cell lung cancer cell line to determine the effect of octreotide and pasireotide. *Results*: Our results showed the traditional somatostatin analog (SSA) octreotide was ineffective in patients with NET G3 and NEC. On the other hand, RT-qPCR showed a high expression of SSTR2 and SSTR5 in H69 cells. Interestingly, while octreotide did not modify H69 cell proliferation, a strong inhibition of proliferation was detected with the use of pasireotide. *Conclusions*: In view of these results, a clinical trial in NET G3 and NEC patients using pasireotide is necessary to determine the usefulness of this drug in improving patient treatment.

## 1. Introduction

Neuroendocrine tumors (NETs) are a rare group of malignancies (0.5% of diagnosed cancers) that derive from neuroendocrine cells and therefore present a highly variable location [1]. NETs have been most frequently described in the gastrointestinal tract and lungs, but have also been located in the central nervous system, thyroid, skin, urogenital system, breasts, and respiratory tract [2]. These tumors show a survival rate of 45% to 55% at five and ten years, respectively [3]. Using the Ki-67 proliferative index (PI), a key prognostic factor for NETs, the World Health Organization (WHO) classified NETs with a Ki-67 PI less than 3% as low-grade (G1), with a ki-67 PI between 3 and 20% as moderate-grade (G2), and with a Ki-67 PI greater than 20% as high-grade (G3) [4]. Unlike low-grade NETs, which are characterized by a low risk of metastasis, G3 NETs are very aggressive tumors associated with rapid proliferation, with a high capacity to generate distant metastases [5,6]. In the latter case, the median survival is approximately 1 year [7]. Surgery is a treatment option in NET G1 tumors. However, at the time of diagnosis of NET G3, the disease frequently presents metastases, so possible treatment options are reduced to palliative therapies to control symptoms [8]. Neuroendocrine carcinomas (NECs) are G3 and include large cell carcinomas and small cell carcinomas, which are very aggressive and have a very bad prognosis, with a median overall survival of one year [9].

The WHO classification for neuroendocrine tumors is divided by location into gastrointestinal and pancreaticobiliary, upper aerodigestive tract and salivary glands, lung and thymus, and thyroid. Within these locations, tumors would be classified by grade into NETs G1, G2, and G3, and NECs [10].

On the other hand, somatostatin (SST), a small peptide that occurs naturally in the body [11], exerts its biological effect through 5 subtypes of somatostatin receptors (SSTR) located in various tissues such as in the lungs, thyroid, immune cells, pancreas, gastrointestinal tract, hypothalamus, or pituitary gland [11]. SST-SSTR binding activates signaling pathways with inhibitory effects including inhibition of cell proliferation and anti-inflammatory effects, among others [12,13,14,15]. These properties make somatostatin a potential candidate for therapeutic use in a wide range of diseases (i.e., acromegaly) [15]. Interestingly, high expression of SSTR2 and SSTR5 was reported in NETs [16], so these receptors and the use of SST have become targets for NET treatment [15,17]. However, the very short half-life of SST (1 to 3 min) was a strong limitation in its therapeutic use [18]. The development of somatostatin analogs (SSA) such as octreotide, the first SSA approved by the Food and Drug Administration (FDA), and lanreotide, both with a longer half-life and high affinity for SSTR2 [14,19], represented a great improvement in the treatment of patients with advanced gastroenteropancreatic NETs. In fact, two different phase 3 clinical trials demonstrated the benefit of using SSA in patients with NETs, which was recommended to delay tumor progression and help with palliative effects [16,20]. In this context, pasireotide, a new second-generation SSA with the same affinity for SST2 as octreotide and lanreotide, but with a high affinity for SSTR1, SSTR3, and SSTR5, was approved by the FDA in 2014 [20,21]. Currently, pasireotide clinical use is focused on the treatment of growth hormone-secreting pituitary tumors and inhibiting corticotropin secretion from pituitary adenoma in patients with Cushing’s disease [22,23]. A phase III clinical trial that used pasireotide in patients with TNE for six months showed no positive results, so it continues to be evaluated at present [19,24].

The aim of this study was to analyze the expression of SSTR in NET G3 and NEC patients, correlating their expression to traditional SSA treatment. In addition, we performed a preliminary in vitro analysis of the possible role of pasireotide in the treatment of NET G3 and NEC.

## 2. Materials and Methods

### 2.1. Patient Data Collection

A total of 20 patients with G3 NETs and NEC and determination of SSTR by nuclear medicine tests were initially recruited at the Medical Oncology Service of the Virgen de las Nieves University Hospital (Granada, Spain). For this purpose, patients were selected by filtering, using the term “octreoscan” (period 2013–2023), selecting all patients with G3 NETs and NEC, and including all patients who met this criterion.

A determination of somatostatin receptor expression in all patients was confirmed by nuclear medicine tests. Most of the patients have been studied using a planar octreoscan (111In-pentetreotide scintigraphy) and some of them have been studied using gallium-PET (68Ga-edotreotide PET), a technique recently incorporated in our hospital. Treatment response has been calculated by RECIST 1.1, defining tumor progression as an increase of 20% or more in tumor lesions size or nuclear medicine uptake and defining a partial response as a decrease of 30% or more in tumor lesions size or nuclear medicine uptake.

The study was approved by the Biomedical Research Ethics Committee of the Andalusian Public Health System in Granada (protocol code 5/23) and conducted in accordance with the Declaration of Helsinki. Written informed consent was obtained for all participants.

### 2.2. Cell Culture

The H69 microcytic lung cancer cell line was provided by the Center for Scientific Instrumentation (CIC) of the University of Granada (Granada, Spain). This cell line was selected for this study because of the high frequency of small cell lung cancer compared with other NETs, G3, and NEC, and the presence of SSTR expression in the bibliography [25,26].

H69 cells were cultured in RPMI-1640 medium supplemented with 10% fetal bovine serum (FBS) (Gibco, Madrid, Spain) and 1% antibiotic (gentamicin/amphotericin-B + penicillin/streptomycin) (Sigma Aldrich, Madrid, Spain). The cultures were maintained in the incubator at 37 °C and a humid atmosphere with 5% CO_2_.

### 2.3. Cell Viability Assay

Cells were seeded at a density of 10^4^ cells/well in 48-well plates in a volume of 300 µl of RPMI. After 24 h, the cells were treated with octreotide and pasireotide. The stock of both drugs was dissolved in dimethyl sulfoxide (DMSO) (Sigma Aldrich, Madrid, Spain) to a final concentration of 28.87 mM and 5 mM, respectively. They were treated with doses of 30, 40, and 50 µM for 5 days. In parallel, controls were carried out with DMSO. After this time, a Cell Counting Kit 8 (CCK8) (AbCam, Cambridge, UK) was performed. Briefly, CCk8 was added to each well at a final concentration of 10% and incubated at 37 °C for 4 h. Finally, absorbance was measured at 450 nm and 620 nm with the Bioteck 800 TS microplate reader (Winooski, VT, USA) and the percent proliferation (PR) was calculated.

### 2.4. RT-qPCR Assay

The expression of SSTR receptors in the H69 cells was determined by RT-qPCR. For this purpose, cells were centrifuged at 250× *g* for 5 min. The cell pellet was resuspended in a Trizol reagent (Sigma-Aldrich, St. Louis, MO, USA). Total RNA was extracted using the RNeasy Mini Kit (Qiagen, Hilden, Germany) and subsequently quantified using the NanoDrop 2000 (ThermoFisher, Waltham, MA, USA). Then, 1 µg of RNA was converted to cDNA using the Retro-transcriptase Kit (Promega, Madison, WI, USA) following the manufacturer’s instructions. Quantitative PCR was performed with SYBR Green (Takara, Kyoto, Japan). Expression of SSTR2 and SSTR5 genes was analyzed. The results were normalized with the endogenous control Glyceraldehyde-3-phosphate dehydrogenase (GAPDH). RT-qPCR was performed on an ABI 7900 system (ABI). Finally, expression levels were calculated by applying the 2^−∆Ct^ method.

### 2.5. Statistic Studies

Statistical analysis was performed with GraphPad Prism 9.4.1 software. All tests were performed in triplicate and the results were expressed as mean ± standard deviation (SD). The statistical analysis used was the one-way ANOVA, a “Z test” comparison for two proportions. A *p*-value of less than 0.05 was considered as statistically significant.

## 3. Results

### 3.1. Clinical Characteristics of Patients

**Figure 1:**
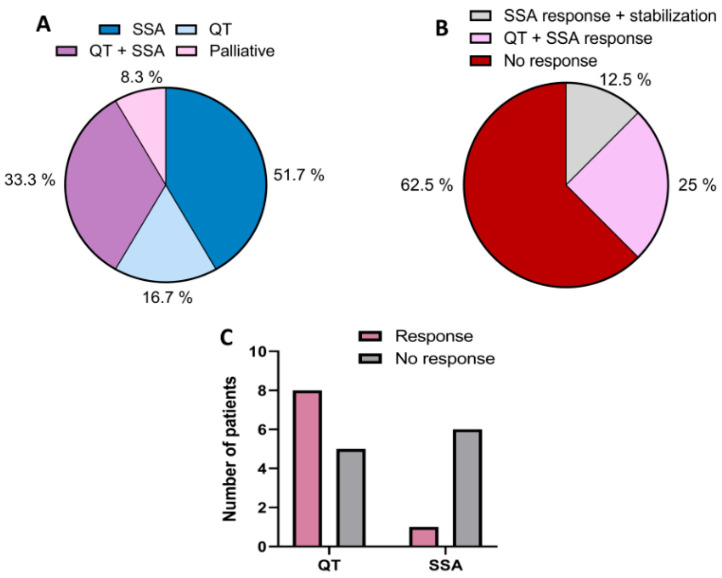
Graphical representation of the clinical data related to the treatment of patients with NET G3 included in the study. (**A**) Treatment of octreoscan- and/or gallium-PET-positive patients with SSA, chemotherapy (QT), chemotherapy together with SSA, or palliative therapy. (**B**) Response of octreoscan- and/or gallium-PET-positive patients to treatments administered. (**C**) Comparison of response or non-response of patients treated with chemotherapy or SSA.

The clinical parameters of G3 NETs and NECs patients who were finally included in the study are summarized in Table 1. Of the 20 patients, 10 (50%) were males and 10 (50%) were females. The mean age was 66 ± 11 years. All patients were diagnosed with G3 NETs and NECs in stage IV, except for one case in stage III, which was tested for somatostatin receptor expression by image studies. The most frequent location of the primary tumor was the lung (25%) and pancreas (20%), followed by the breast (15%) and cervix (15%). The remaining patients (25%) presented other variable locations of the primary tumor (Table 1). SSTR expression was analyzed by octreoscan and/or gallium-PET. Most of the patients (60%) were octreoscan-positive, except one, which was gallium-PET-positive. In addition, 50% of the positive patients had small cell neuroendocrine carcinomas (G3), and the other 50% had non-small cell NET G3. Of the positive patients, 51.7% were treated with SSA, such as octreotide, lanreotide, or lutetium (177Lu-DOTATATE), 16.7% with chemotherapy, 33.3% with chemotherapy and SSA, and the remaining 8.3% with palliative therapies (Figure 1A).

The response of SSTR expression-positive patients treated with SSA alone or SSA combined with chemotherapy was analyzed, finding that 62.5% showed no response to the treatment administered. Only 25% of patients treated with chemotherapy in combination with SSA showed a positive response and the 12.5% treated with SSA alone showed either a response or disease stabilization (Figure 1B). On the contrary, patients treated with chemotherapy alone responded 61.5% to treatment. A comparison between SSA and chemotherapy treatment showed that 8 patients responded versus 5 patients who did not respond after chemotherapy, and that one patient responded versus 5 patients who did not respond after SSA treatment (Figure 1C). The “Z test” for the comparison of the two independent sample proportions showed a significant *p*-value (0.039) in relation to QT and SSA treatment.

### 3.2. In Vitro Cell Viability after Pasireotide Treatment

**Figure 2:**
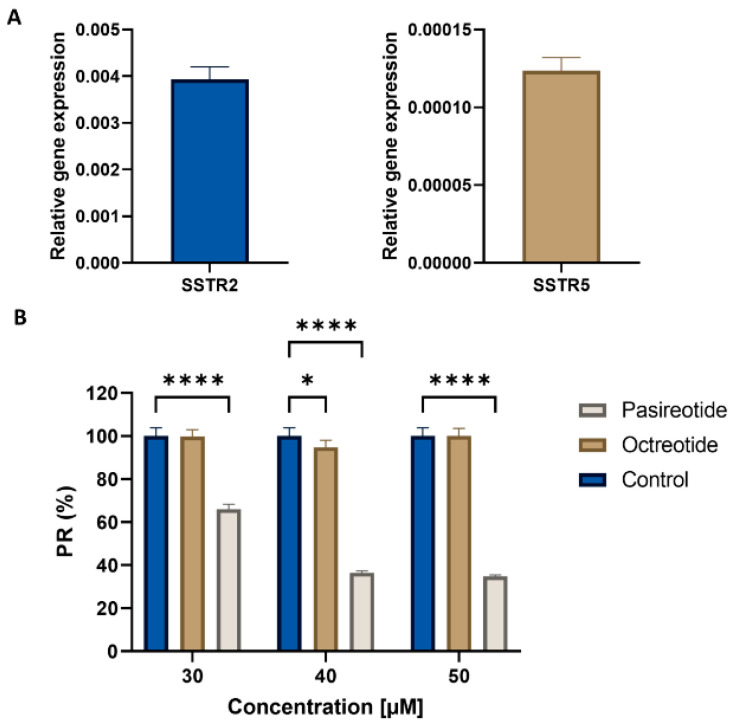
Assay of Pasireotide effect on H69 cell line. (**A**) SSTR2 and SSTR5 somatostatin receptor expression in the H69 cell line. (**B**) Graphic representation of H69 cell relative proliferation after treatment with pasireotide and octreotide. Data represents the mean value ± S.D. * *p* < 0.05. **** *p* < 0.0001.

The H69 small cell lung cancer cell line was selected to carry out a preliminary study of the pasireotide effect. Previously, RT-qPCR analysis showed a high expression of SSTR2 and SSTR5 somatostatin receptors in this cell line (Figure 2A). Pasireotide treatment inhibited the viability of H69 cells in a dose-dependent manner, showing IC50 values of 35.4 µM. By contrast, exposure of H69 cells to octreotide did not induce any modulation of cell viability, even when a concentration of 300 µM was used (Figure 2B).

## 4. Discussion

Our results showed that a large proportion of selected patients with NET G3 and NEC expressing SSTR receptors did not respond to traditional SSA treatment. The CLARINET study demonstrated the therapeutic benefit of a new SSA called lanreotide in patients with metastatic enteropancreatic NET G1 or G2 (Ki67 < 10%) [16]. It should be noted that these clinical trials were conducted in patients with NET G1-2, and had not been demonstrated in NET G3 [16,20]. Our study compared the response of traditional SSA with chemotherapy in NET G3. Interestingly, chemotherapy achieved a greater tumor response (61.5%) than SSA (12.5%) in octreoscan/gallium-PET-positive patients, which allows us to hypothesize the presence of a resistance mechanism against traditional treatment with SSAs. It should be noted that both octreotide and lanreotide show high binding affinity to SSTR2. However, the expression of SSTR receptor subtypes is highly variable, depending on the tissue and tumor type [11]. Therefore, the low antitumor effect may be related to the expression of receptors other than SSTR2. Furthermore, some studies demonstrated that both octreotide and lanreotide increased SSTR5 expression [27]. This contradictory effect of overexpressing SSTR5 and inhibiting SSTR2 may be responsible for the lack of response of traditional SSAs.

On the other hand, the lung is the most frequent location of primary NETs and NECs (approximately 20%) [28,29], which was consistent with our study where a lung primary tumor was detected in 25% of cases. Because the largest subgroup of neuroendocrine neoplasms consisted of small cell lung carcinoma (SCLC) (mitotic count (MC) >10 in 2 mm^2^), the H69 cell line (small cell lung carcinoma) was selected for in vitro assays [29,30]. Interestingly, H69 cells showed high SSTR2 and SSTR5 expression, supporting previous results of Kaemmerer et al. [25] and King et al. [26]. First, exposure of H69 cells to octreotide did not show any antiproliferative effect, supporting the data from Exner et al. in five pancreatic, colon, and lung NET cell lines, in which this drug did not show any activity [31].

By contrast, the use of pasireotide, a new SSA analog with a wide range of SSTR receptor affinity (SSTR2, SSTR1, SSTR3, and SSTR5), showed a strong antiproliferative effect in H69 cells. In fact, in vivo pituitary tumors expressing low levels of SSTR2 and high levels of SSTR5 showed a greater therapeutic response with the use of pasireotide versus octreotide [32].

Therefore, these results suggest that differential expression of the somatostatin receptor may be the cause of traditional SSA analog failure in patients. Studies according to SSTR expression in NETs and NECs expose different basal expressions. Tsuta et al., expose that there are few patients with NETs and NECs who express SSTR5 [33]. However, Muscarella et al. have found SSTR5 expression in NEC circulating cells in the majority of studied patients [34]. Qian et al. report that 43% of NETs and NECs have an expression of SSTR5 [34]. Moreover, Wang et al. published that high-grade NETs and NECs from gastrointestinal origins have a positivity of 34%for SSTR5 [35]. In small cell lung cancer, Lapa et al. have described a positivity of 15% for SSTR5 [36]. All these articles demonstrate that there are patients in different types of NETs and NECs that have SSTR5 expression, which could explain the resistance to traditional SSA and could be an important therapeutic opportunity for these patients.

However, this study has several limitations in being considered for future investigations. On the one hand, the number of patients included in the study is small, which is due to the low incidence of NECs and G3 NETs compared to other tumor types and the low percentage of high-grade neuroendocrine tumors patients with SSR determination by nuclear medicine tests. On the other hand, only one cell line has been used to test the efficacy of pasireotide (H69), and it would be interesting for future studies to include a greater number of cell lines from various tumor locations in order to increase the robustness of the results.

## 5. Conclusions

In this study, we demonstrate the ineffectiveness of a traditional SSA, such as octreotide, in NET G3 and NEC patients in terms of therapeutic response, despite the expression of somatostatin receptors in octreoscan/gallium-PET. Our in vitro assays using the H69 cell line are consistent with clinical data regarding the ineffectiveness of octreotide as therapy against NEC. By contrast, the new SSA analog named pasireotide demonstrated a strong antiproliferative effect in these tumor cells, characterized by a high expression of SSTR2 and SSTR5. This effect could be correlated with the higher range of affinity of this drug to different SSTRs versus octreotide. Therefore, our results support interest in carrying out a clinical trial using pasireotide in the treatment of NET G3 and NEC as a new strategy to improve the therapeutic response of these tumors, previously histologically analyzing the presence of SSTR5 in candidate patients in a personalized approach.

## Figures and Tables

**Table 1:** Characteristics and clinical data of patients.

Patient	Age	Sex	Octreoscan (111In-Pentetreotide Scintigraphy) (Planar) or Gallium-PET (68Ga-Edotreotide PET)	Primary Tumor Localization	Stage	Tumor Grade	Ki67	Treatment	Treatment Response (RECIST 1.1)
1	59	F	Negative *	Lung NEC	IV	G3 (large cell)	Unknown	QT	PR
2	53	M	Positive *	Pancreas NEC	IV	G3 (small cell) + G1 (differentiated focuses)	1–20%	Lanreotide	CR
3	75	F	Positive *	Gallbladder NEC	IV	G3 (small cell)	70%	Palliative	Unknown
4	58	F	Positive (weak positivity) *	Breast NEC	IV	G3 (small cell)	80–90%	QT	PD
5	54	F	Positive *	Cervix NET	IV	G3	90%	177Lu-DOTATATE	SD
6	81	F	Negative *	Cervix NEC	IV	G3 (large cell)	Unknown	Palliative	Unknown
7	79	M	Positive **	Cervical NEC	III	G3 (large cell)	90%	QT + radiotherapy	CR
8	78	M	Negative *	Colon NET	IV	G3	80%	QT	PD
9	76	F	Negative *	Pancreas NET	IV	G3	40%	QT	PR
10	61	F	Positive *	Rectum NEC	IV	G3 (small cell)	80%	QT + Lanreotide	PD
11	51	M	Negative *	Retroperitoneal NEC	IV	G3 (small cell)	60–70%	QT	PD
12	54	F	Negative *	Cervix NET	IV	G3	80%	QT	CR
13	65	M	Positive *	Lung NET	IV	G3	Unknown	QT + Octreotide	PD
14	69	F	Positive *	Breast NET	IV	G3	70–80%	Lanreotide	PD
15	78	M	Positive (weak positivity) *	Microcytic lung cancer (NEC)	wd	G3 (small cell)	Unknown	Octreotide	PD
16	70	M	Positive (weak positivity) *	Microcytic lung cancer (NEC)	wd	G3 (small cell)	>20%	QT + octreotide	PR
17	70	M	Negative *	Microcytic lung cancer (NEC)	wd	G3 (small cell)	Unknown	QT	PR
18	65	F	Positive *	Breast NET	IV	G3	35%	Octreotide	PD
19	56	M	Positive *	Pancreas NET	IV	G3	Unknown	QT + Lanreotide	SD
20	80	M	Negative *	Ampulla of vater NET	IV	G3	>40%	QT	PR

Widespread disease (wd); chemotherapy (QT); female (F); male (M); * octreoscan (planar); ** gallium-PET; partial response (PR); complete response (CR); progressive disease (PD); stable disease (SD).

## Data Availability

Data presented in the study are available upon request from the corresponding author.
